# Polymorphism and Balancing Selection of MHC Class II DAB Gene in 7 Selective Flounder (*Paralichthys olivaceus*) Families

**DOI:** 10.1155/2011/613629

**Published:** 2011-06-22

**Authors:** Min Du, Song-lin Chen, You Liang, Lei Wang, Feng-tao Gao, Yang Liu, Xiao-Lin Liao

**Affiliations:** ^1^Key Lab for Sustainable Utilization of Marine Fisheries Resources, Ministry of Agriculture, Yellow Sea Fisheries Research Institute, Chinese Academy of Fishery Sciences, Nanjing Road 106, Qingdao 266071, China; ^2^College of Aqua-life Science and Technology, Shanghai Ocean University, Shanghai 200090, China; ^3^Honghe University, Yunnan Province, Mengzi, 661100, China

## Abstract

In order to determine the genetic variation of the MHC class IIB exon2 allele in the offspring, 700 fry from seven families of Japanese flounder challenged with *V. anguillarum* were studied, and different mortality rates were found in those families. Five to ten surviving and dead fry from each of the seven families were selected to study the MHC class II B exon2 gene with PCR and a direct sequencing method. One hundred and sixteen different exon2 sequences were found and 116 different alleles were identified, while a minimum of four loci were revealed in the MHC class II B exon2 gene. The ratio (*d*_*N*_/*d*_*S*_) of nonsynonymous substitution (*d*_*N*_) to synonymous substitutions (*d*_*S*_) in the peptide-binding region (PBR) of the MHC class IIB gene was 6.234, which indicated that balancing selection is acting on the MHC class IIB genes. The MHC IIB alleles were thus being passed on to their progeny. Some alleles were significantly more frequent in surviving than dead individuals. All together our data suggested that the alleles Paol-DAB*4301, Paol-DAB*4601, Paol-DAB*4302, Paol-DAB*3803, and Paol-DAB*4101 were associated with resistance to *V. anguillarum* in flounder.

## 1. Introduction

Genes of the major histocompatibility complex (MHC) are characterized by extremely high levels of polymorphism in cell surface glycoprotein class I and II molecules. They play a primary role in both innate and adaptive immunity by presenting self- and foreign peptides to T cells (CD4^+^ T cells or CD8^+^ T cells) [1] in vertebrate organisms, and subsequently initiate a specific immune response [2].

Unlike the case in mammals, MHC class I and class II regions in teleost fish are situated on different linkage groups and therefore do not form a complex [3–5]. MHC genes in fish have received considerable attention since the first teleost fish MHC gene fragments were isolated from carp (*Cyprinus carpio L.*) by Hashimoto et al. [6]. MHC class I and class II both contain a peptide-binding region (PBR). The exon2 sequence of the MHC class II B gene is known to cover the majority of the polymorphism and has been considered a candidate molecular marker for an association between these alleles and resistance/susceptibility to various diseases [7]. There are reports of polymorphism of exon2 of MHC class II B gene in a number of vertebrates, including mammals [8, 9], reptiles [10, 11], amphibians [12], and fish [13–15].

It is believed that balancing selection maintains this large variation, which includes frequency-dependent selection, over dominant selection, and positive selection across habitats, but the exact nature of the selection process continues to be a topic of debate [16–18].

Japanese flounder (*Paralichthys olivaceus*) is an economically important marine fish in China, and a few studies have been reported on the MHC class II B gene [15, 19, 20]. For example, Srisapoome et al. [19] reported the expression level of MHC II B cDNA. Zhang et al. [20] studied polymorphism in the flounder MHC class II B gene. Xu et al. [15] demonstrated an association between MHC class II B exon2 and resistance to *V. anguillarum* in 60 families of Japanese flounder, and thus the alleles associated with resistance and susceptibility to* V. anguillarum* were discovered. 

In order to breed a new flounder strain with enhanced disease resistance and growth rate, selective breeding has been carrying out, since 2002. Three basic populations (i.e., Japanese (JS), Resistance (RS), and Yellow Sea (YS) populations) were developed in 2002 and 2003 [21]. JS were imported from Japan in 2003; RS were obtained from natural selection and artificial challenge with *Vibrio anguillarum*; YS were captured from the Yellow Sea in 2003. These were called “generation 0” (G_0_). A little more than three years later, in March, 2007, the fry of the three basic populations that had become sexually mature were selected to mate and produced 63 full-sib families or half-sib families and were designated “generation 1” (G_1_). After artificial challenge with *Vibrio anguillarum*, the survival percentage ratios (Mean ± S.D. (%)) of the families studied (family 0751, family 0768, family 0743, family 0750, and family 0719) were 54.13 ± 1.23, 62.08 ± 22.52, 7.27 ± 3.57, 64.05 ± 0.74, and 30.86 ± 7.22, respectively; the survival ratio of the resistance families was not available. Two years later, in March, 2009, sexually mature fish were selected for mate (generation 2 (G_2_)) and artificial challenge were performed, with the result that the survival ratios of the families were different. 

The fry of the next generation exhibited clear genetic information within each family. In this study, their offspring were infected with *V. anguillarum* and their survival rate was recorded. We amplified and directly sequenced MHC class II B exon2 in order to estimate the number of MHC class II loci, assess the MHC polymorphism of selected individuals, and test for balancing selection, as well as to discover the pattern of the inheritance of the allele in seven families of Japanese flounder.

## 2. Materials and Methods

### 2.1. Experimental Design

**Figure 1:**
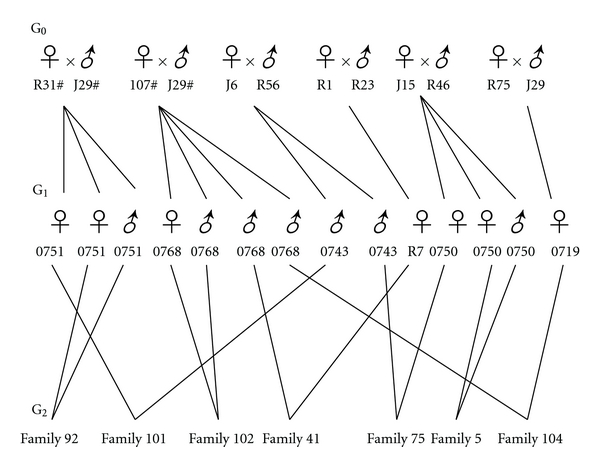
The pedigree denotes the families in generation G_2_ with parents in G_1_ and grandparents in the G_0_.

According to a previous study [15], the fish from family 0768 and family 0751 had the Paol-DAB*4301 allele, while the fish from the 0743, R7, 0750, and 0719 families did not. The Paol-DAB*4301 allele in flounder was associated with resistance to *V. anguillarum*. The survival ratio of families 0768 and 0751 was higher than that of the 0743, R7, 0750, and 0719 families. Males and females from G_1_ in these six families were selected as parental fish to propagate the offspring in G_2_ (Figure 1). The brood fish in G_0_ were involved in our previous study [21]. Families 92, 102, and 5 were offspring of self-cross of families 0751, 0768, and 0750, respectively, (Figure 1). Family 101 had one dam from family 0743 crossed with one sire from family 0751. Family 41 had a dam from family R7 crossed with one sire from family 0768. Family 75 were the offspring of one dam from family 0750 and one sire from family 0743, and family 104 were the offspring of one dam from family 0719 and one sire from family 0768. A total of 7 full-sib families of Japanese flounder were established, as reported by Chen et al. [21] and were reared at the fish farming factory at Haiyang city, Shandong province. The fry were fed a commercial diet and were kept in separate tanks.

### 2.2. Challenge Experiment

Approximately 100 individuals from each family and a total of 700 offspring from 7 full-sib families were used in the challenge experiment. The body weight of the fish analyzed was 12–17 g. The test fish of each family were kept in a separate tank at the same farming factory under flow-through conditions with a fresh water supply at 20 ± 0.5°C and were fed twice daily. The *V. anguillarum *isolated by our laboratory was used in the challenge test and prechallenge experiment, and the median lethal concentration was determined according to Xu et al. [15]. Dead fish were recorded and collected every day. The challenge experiment was terminated 14 days after infection. The survival ratio (Mean ± S.D. (%)) of families 5, 41, 102, 75, 101, 92, and 104 was 78.3 ± 7.43, 32.2 ± 3.61, 31.9 ± 22.36, 37.9 ± 9.44, 33.4 ± 3.7, 21.8 ± 11.97, and 55.6 ± 1.83, respectively. In addition to the daily recording of the fish that had died, fin clippings were taken from all fish and were stored individually in absolute ethanol.

### 2.3. Sampling and DNA Isolation

Fin samples from the top 10 (families 5, 41, 75, 92, and 102, resp.) or 20 (families 101, 104, resp.) individuals to die and the top 10 (families 5, 41, 75, 92, and 102, resp.) or 20 (families 101 and 104, resp.) surviving individuals from each family were collected from the challenge trials (Table 1). Table 1 shows the number of the dead fish. Surviving individuals were selected from the seven families of Japanese flounder in the study. Genomic DNA was extracted from the dorsal or caudal fin tissue of the dead and surviving fry via a modified phenol-chloroform method [22]. The integrity of the DNA was analyzed on a 1% agarose gel containing 0.5% *μ*g/mL ethidium bromide by electrophoresis and visualized under UV light. The concentrations of DNA were measured using a GENEQUANT Pro (Pharmacia Biotech Ltd.) RNA/DNA spectrophotometer. Finally, DNA was adjusted to 100 ng/*μ*L and stored at −20°C.

### 2.4. Primer Design and Polymerase Chain Reaction (PCR)

Two oligonucleotides of the gene-specific primers: fMPN (5-CTCCCTCTTCTTCATCACGG T-3) and fMPC (5-ACACACTCACCTGACTTCGT-3) were used for amplifying the flounder MHC II B sequences, which were designed according to the flounder cDNA sequences reported [20] and published communications [15]. The forward primer for class II B is based on the end of exon1 sequence, and the reverse primer for class II B is on the end of exon2 sequence, respectively. Polymerase chain reaction (PCR) was performed in a total volume of 25 *μ*L, which consisted of 100 ng template DNA, 2.5 *μ*L of 10 × *Taq* polymerase buffer (*TransGen Biotech*), 1.5 mM MgCl_2_, 0.2 mM deoxynucleotide triphosphate, 0.2 *μ*M each of the forward and reverse primers, and 1 unit of *Taq* polymerase (*TransGen Biotech*). The amplification conditions were optimized to reduce the nonspecific amplification [23]. Thermocycling was conducted on a Peltier Thermal Cycler (PTC-200) and the amplification schedule was 94°C for 5 minutes, 30 cycles of 94°C for 40 s, 52°C for 40 seconds, 72°C for 50 seconds, and finally 72°C for 10 minutes. The Molecular Imager Gel Doc XR system (*Bio-rad*) was used to check for integrity and visualize the PCR products by electrophoresis on a 1% agarose gel. The amplified fragments exhibited one distinct band with an approximate length of 500 bp.

### 2.5. Cloning and Sequencing

PCR products were excised and then purified with the QIAEX II gel extraction kit (*Qiagen*). According to the standard protocol, the purified products were ligated into a PBS-T vector with a TA cloning kit (*Takara*) and then cloned into TOP 10 *Escherichia coli* competent cells (*TransGen Biotech*). Positive clones were screened in PCR reactions with the cloning primers T7 and M13R. The PCR products for appropriately sized clones were cleaned with a Qiaquick PCR purification column (Qiagen) before cycle sequencing with a Big Dye Terminator cycle-sequencing kit following the manufacturer's instructions on an ABI 3730 automated sequencer (Applied Biosystems, Foster City, CA, USA). All of the alleles shown in the present study were confirmed by sequencing a minimum of five times in ten individuals from five of the families, but up to 10 times or more in twenty individuals from two of the families.

### 2.6. Genotyping, Sequence Analysis, and Statistical Tests

MHC gene sequences were aligned using DNAMAN software. Comparison of these nucleotide sequences and deduced amino acid sequences was performed using the MEGA4.0 program [24]. The relative rates of synonymous (*d*_*S*_) and non-synonymous (*d*_*N*_) substitution were determined according to Nei and Gojobori [25] and corrected for multiple hits (Jukes and Cantor) [26] using MEGA4.0. The frequency of polymorphism was analyzed using all of the alleles in the program by means of DnaSP4.0 [27] and DAMBE [28] with Jukes-Cantor distances. Statistical analysis was obtained using SPSS13.0. Allele frequency discrepancies were verified using Fisher's exact test and the significance level [29] was determined for every individual (*n* = 180) and every family (*n* = 7).

## 3. Results

The average mortality ratio was 66.65 ± 24.31 (the Mean ± S.D. (%)), which was calculated 14 days after the bacterial infection in the 7 families. In this study, we verified 116 distinct MHC class II nucleotide sequences from 180 individuals of the seven flounder families (Table 1). Among these sequences, 72 sequences were present only once, and 17 sequences were the same as in previous reports [15], that is, Paol-DAB*0101, Paol-DAB*0301, Paol-DAB*0801, Paol-DAB*0901, Paol-DAB*2201, Paol-DAB*3201, Paol-DAB*3501, Paol-DAB*3801, Paol-DAB*3803, Paol-DAB*3804, Paol-DAB*3805, Paol-DAB*4302, Paol-DAB*0102, Paol-DAB*2202, Paol-DAB*0502, Paol-DAB*4101, and Paol-DAB*4301. 99 sequences were newly discovered in the present study and were deposited in GenBank (accession no. HQ634973–HQ635071; Table 2). 

The new alleles detected in this study were based on the deduced amino acid sequences and designated based on the rules reported previously [15, 30–32]. For example, in Paol-DAB*0103, Paol refers to *Paralichthys olivaceus*, D to class II, A to the family designation, and B to the *β* chain-encoding genes. In the four digits after the asterisk, the first two digits refer to the major type (i.e., alleles that differ by at least five amino acid substitutions), and the last two digits to the subtype (i.e., alleles that differ by less than five amino acid substitutions within a single major type). 

### 3.1. MHC Class II B Sequence Diversity

The length of the amplified MHC class II sequence was 407 bp. The sequences covered 91 amino acid residues of the MHC class II B exon2 and complete intron1 (84/96 bp, including a 12 bp repeat loci) [15]. There were no frame-shift mutations or stop codons in these alleles. There were 151 polymorphic sites across the 116 different MHC class II exon2 sequences. The average number of nucleotide differences (*k*) was 20.84, and the nucleotide diversity value (*P*_*i*_) for these sequences was 0.07634. Among the individuals of the seven families, five (10 individuals from families 5, 41, 75, 92, 102, resp.) or ten (20 individuals from family 101, 104, resp.) clones per individual were sequenced. Only one sequence was present in five clones per individual from 25 individuals of families 102, 92, 75, 41, and 5; two sequences were detected in five or ten clones per individual from 71 individuals; three sequences were found in five or ten clones per individual from 57 individuals; four sequences were present in five or ten clones per individual from 18 individuals; five sequences were only detected in ten clones per individual from 5 individuals from family 101 and family 104; six sequences were only detected in ten clones per individual from 3 individuals from family 101; seven sequences were detected in ten clones per individual from one individual from family 101, indicating that this primer set amplifies at least four loci or copies in this species (Table 3) [6, 33].

**Figure 2:**
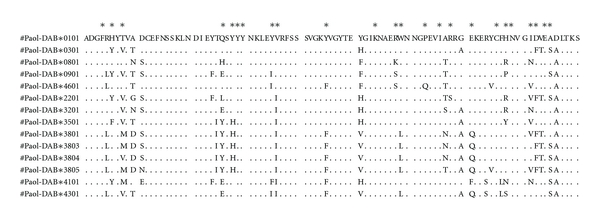
Putative amino acid sequences for MHC II B exon2 alleles of Japanese flounder. Dots denote identity with the first sequences; the putative peptide binding region is indicated with asterisks.

The putative amino acid sequences for the MHC II B exon2 alleles of Japanese flounder along with those previously reported are shown in Figure 2. The nucleotide sequence homology in the MHC class II B genes ranged from 87% to 99%. Alleles differed in amino acid composition by one to twenty-eight substitutions out of 91 sites.

### 3.2. Alleles Distribution in the Seven Flounder Families

Alleles were shared among certain individuals and families in this study.  Table 4 summarizes the alleles which were frequently present in individuals of the seven families investigated (one allele Paol-DAB*4601 was presented only in this study, while the other 16 alleles have been reported previously [15], and these were Paol-DAB*4301, Paol-DAB*4302, Paol-DAB*0101, Paol-DAB*3201, Paol-DAB*2201, Paol-DAB*3803, Paol-DAB*3804, Paol-DAB*0102, Paol-DAB*0301, Paol-DAB*4101, Paol-DAB*0801, Paol-DAB*2202, Paol-DAB*0901, Paol-DAB*3805, Paol-DAB*0502, and Paol-DAB*3501, resp.). Thus, four alleles (Paol-DAB*4301, Paol-DAB*0101, Paol-DAB*3201, and Paol-DAB*2201) were obtained from three hundred and ninety-seven clones from forty juveniles from family 101, with a frequency of 16%, 22.1%, 20.3%, and 22.6%, respectively. Five alleles (Paol-DAB*4301, Paol-DAB*4601, Paol-DAB*3803, Paol-DAB*0101, and Paol-DAB*0102) were found to be present in three hundred and ninety-five clones from forty juveniles from family 104, with a frequency of 19.9%, 20.2%, 16.4%, 19.9%, and 7.3%, respectively. Two alleles (Paol-DAB*0301 and Paol-DAB*4101) were obtained from one hundred clones from twenty juveniles from family 5 with a frequency of 6% and 34%. Four alleles (Paol-DAB*0101, Paol-DAB*0801, Paol-DAB*3803, and Paol-DAB*2202) were present in one hundred and five clones from twenty juveniles in family 41 with a frequency of 21.9%, 46.7%, and 22.9%, respectively. Four alleles (Paol-DAB*0901, Paol-DAB*2201, Paol-DAB*3805 and Paol-DAB*0502) were obtained from one hundred and two clones from twenty juveniles in family 75, with a frequency of 25.5%, 22.6%, 20.6%, and 15.7%, respectively. Two alleles (Paol-DAB*3501 and Paol-DAB*4301) were obtained from one hundred and one clones from twenty juveniles family 92 with a frequency of 40.5% and 43.6%, and three alleles (Paol-DAB*0101, Paol-DAB*4301, and Paol-DAB*3803) were presented in ninety-seven clones in twenty juveniles from family 102 with a frequency of 39.2%, 24.7%, and 13.4%, respectively.

### 3.3. Association between Allele Frequency and Resistance/Susceptibility to *V. anguillarum* in the Surviving and Dead Individuals within the Seven Families

Most of the 116 alleles were presented only once or twice and therefore they were excluded from the analysis of the association with bacterial resistance. Fourteen alleles were selected for further analysis, of which one allele, Paol-DAB*4601, was first identified in this study, while the 13 other alleles were identified in previous reports [15]. The latter were Paol-DAB*0101, Paol-DAB*0301, Paol-DAB*0801, Paol-DAB*0901, Paol-DAB*2201, Paol-DAB*3201, Paol-DAB*3501, Paol-DAB*3801, Paol-DAB*3803, Paol-DAB*3804, Paol-DAB*3805, Paol-DAB*4101, and Paol-DAB*4301. A sharing of the same alleles, Paol-DAB*4301 and Paol-DAB*0101, were observed in four of the seven families examined (Table 4), with the frequency different in each family. In family 104, there was a 7.6% frequency in the surviving individuals and a 12.3% frequency in dead individuals for Paol-DAB*4301, as well as a 10.8% frequency in the surviving individuals and a 9.1% frequency in dead individuals for Paol-DAB*0101. In family 92, there was a 24.8% frequency for Paol-DAB*4301 in the survivors and an 18.8% frequency in the dead individuals. In family 101, there was an 11.2% frequency for Paol-DAB*4301 in survivor individuals and a 4.8% frequency in dead individuals, and this difference was significant (*P* = 0.0010); there was a 10.2% frequency found for Paol-DAB*0101 in the surviving individuals and an 11.8% frequency in the dead. In family 102, there was a 12.4% frequency in the surviving and 12.4% frequency in the dead individuals for Paol-DAB*4301, as well as a 17.5% frequency in the survivors and a 21.7% frequency in dead individuals for Paol-DAB*0101. In family 41, an 11.4% frequency was found in the surviving individuals while a 10.5% frequency was found in the dead individuals for Paol-DAB*0101. Some MHC class II B allele frequencies differed significantly between the surviving and dead individuals within the family. In family 104, the Paol-DAB*4601 allele, which was newly identified in this study, was significantly more frequent in the surviving (13.6%) individuals than in the dead individuals (6.5%, *P* = 0.001).

In family 101, the Paol-DAB*2201 frequency in the surviving individuals (8.4%) was significantly lower than in the dead individuals (14.2%, *P* = 0.001), while in family 41, the Paol-DAB*3803 allele was significantly more frequent in the survivors (17.1%) than the dead (5.7%, *P* = 0.009). In family 5, the Paol-DAB*4101 allele was significantly more frequent in the surviving (26%) than dead fish (8%, *P* = 0.01), while the Paol-DAB*0301 allele was significantly more frequent in the dead (38%) than the survivors (21%, *P* = 0.008). In family 75, family 92, and family 102, the difference between the allele frequencies in the surviving and dead individuals was not significant. These results suggested that the Paol-DAB*4301, Paol-DAB*4601, Paol-DAB*4302, Paol-DAB*3803, and Paol-DAB*4101 alleles were associated with resistance to *V. anguillarum, *while Paol-DAB*2201 and Paol-DAB*0301 appeared to be associated with susceptibility to this bacteria.

### 3.4. Evidence for Balancing Selection

The pattern of nucleotide substitution was examined in the putative PBR (peptide-binding region) and other regions. Twenty-three amino acid residues were selected as the putative PBR sites in the human regions [34]. The mean numbers of synonymous substitutions per synonymous site (*d*_*S*_) and nonsynonymous substitutions per nonsynonymous site (*d*_*N*_) were based on pairwise comparisons among the whole sequences in seven families (Table 5). In the putative PBR region, the mean *d*_*N*_ (0.231, 0.134, 0.109, 0.180, 0.167, 0.146, 0.147, and 0.177) was significantly higher than the mean *d*_*S*_ (0.037, 0.031, 0.048, 0.060, 0.024, 0.009, 0.024, and 0.004) for all of the pairwise comparisons, respectively. Furthermore the *d*_*N*_/*d*_*S*_ in the PBR (6.243, 4.323, 2.271, 3.000, 6.96, 16.2, 6.125, 44.25) was greater than that in the non-PBR (1.390, 2.5, 1.109, 1.065, 1.27, 1.410, 1.606, 1.389) in terms of the whole sequence and in each family sequence, respectively. These results indicated that positive selection was at work in the PBR of MHC class II B genes.

### 3.5. Inheritance of the Allele in the Next Generation

The pedigree of the Japanese flounder was shown in Figure 1. At G_1_, both family 0768 and family 0751 had Paol-DAB*4301 alleles, while family 0768 also had Paol-DAB*0801 allele. We found that the Paol-DAB*4301 alleles were presented in families 101, 104, 92, and 102, and Paol-DAB*0801 in family 41 at G_2_, respectively. The sire and dam of family 92 were from family 0751, while the sire and dam of family 101 were from family 0743 and family 0751, respectively. The sire and dam of family 102 were from family 0768. The sire and dam of family 41 were from family 0768 and family R7, respectively, while the sire and dam of family 104 were from family 0768 and family 0719, respectively. This denoted that the MHC II B alleles were passed on to the progeny. Neither Paol-DAB*4301 nor Paol-DAB*0801 was present in family 75 and 5. The sire and dam of family 75 were from family 0743 and 0750, respectively, while the sire and dam of family 5 were both from family 0750. The distribution patterns of the alleles in each family were obtained from DNA sequence analysis and are shown in Table 4.

## 4. Discussion

The major histocompatibility complex (MHC) is a vital portion of the vertebrate immune system, and MHC allele diversity is critical for resistance against parasites [14]. Dixon et al. [35] discovered 57 alleles in 17 individuals with greater polymorphism than is found in most mammals. This region was selected for amplification as a result of it covering the whole of exon2 in the *β*1 domain, which corresponded to the highly variable region of the PBR. Therefore, in this study, we investigated variations in seven flounder families using MHC class II B exon2 as a gene marker, and the diversity was found to differ significantly (116 sequences in 180 individuals). At least four MHC class II exon2 loci were present in Japanese flounders, which was more than the number previously reported by Xu et al. [15] and Zhang et al. [20]. Homology of these alleles from each individual was from 89% to 100%, and in all the individuals examined was from 87% to 100%, with levels as high as 0.11 in mammals [36].

In a previous study by Xu et al. [15], Paol-DAB*4301, Paol-DAB*0601, Paol-DAB*0801, Paol-DAB*2001, and Paol-DAB*3803 were the alleles which found to be associated with resistance to *V. anguillarum, *while Paol-DAB*1601, Paol-DAB*2201, and Paol-DAB*2701 were the alleles which associate with susceptibility. In the present study, we found that Paol-DAB*4301, Paol-DAB*4601, Paol-DAB*3803, and Paol-DAB*4101 were associated with resistance to *V. anguillarum,* while Paol-DAB*3201, Paol-DAB*2201, and Paol-DAB*0301 alleles were associated with susceptibility. Moreover, the significant difference in the frequency of each allele between the survivors and dead fish was only found in one family. 

In addition to the fact that analysis within family was less influenced by the background of the families' genetic variations. The link between the alleles and the bacterial resistance was unpredictable both within and among families, as well as the pooled material. It might be that the alleles are indirectly involved in the resistance to pathogens, or it was possible that the families which were challenged displayed different but “functionally similar” alleles by chance.

Xu et al. [15] demonstrated that the MHC II B alleles were passed on to the progeny. In the present study, the allele Paol-DAB*4301 in family 0768 at G_1_ was also discovered in families 101, 104, 92, and 102 at G_2_. This stability of inheritance within the families had been shown for two generations. Klein [37] reported that the high levels of allelic diversity and polymorphism in the MHC resulted from the long-term coevolution of parasites and MHC molecules. In this study, no complete sequences (alleles) were shared across all of the families, while certain alleles were shared among individuals and two to three families in the Japanese flounder. The sequences of the MHC alleles were not consistent with the phylogeny relationships of individuals seen as a family. This was in agreement with the result of Ye et al. [38], who reported that the MHC allele sequences were not consistent with the phylogeny relationships of a population in a closely related species. Therefore, to fully understand the polymorphism of the MHC class II genes in Japanese flounder, it was necessary to carry further studies, including an estimation of the number of gene loci, introductions of improved methods, and analysis of a greater number of individuals as well as genes and functions. Genetic polymorphism of MHC was generally thought to be maintained by a balancing selection driven by host-parasite coevolution [39–42]. Evidence for balancing selection operating in the MHC class II B gene was a significantly higher rate of non-synonymous mutation (*d*_*N*_/*d*_*S*_> 1), which indicated that the rate of non-synonymous substitution per non-synonymous sites exceeds that of synonymous substitution per synonymous sites [43, 44].

We examined the *Paol-DAB* alleles, including both the whole sequences and the sequences in each family discovered in the present study and found that the *d*_*N*_/*d*_*S*_ ratio (6.234, 4.323, 2.271, 3.000, 6.96, 16.2, 6.125, and 44.25, resp.) in the putative PBR regions was higher than that of *d*_*N*_/*d*_*S*_ (1.390, 2.5, 1.109, 1.065, 1.27, 1.410, 1.606, and 1.389, resp.) in the non-PBR regions in the MHC class II exon2 domain of Japanese flounder (Table 5), as was also the case for the human, nonhuman primate, and mouse class II genes[44–46]. This was evidence for balancing selection or positive selection at work in the PBR of MHC class II B genes. In this study, certain alleles exhibited a high frequency in individual families (Table 4), while other alleles were found only once or twice in seven families, which indicated frequency-dependent selection [17, 47], that is, one model of balancing selection, acting on the polymorphism of the MHC class II B genes in the Japanese flounder.

In the seven families investigated, the percentage of heterozygosity (two different sequences in one individual) in families 101, 104, 102, 92, 75, 41, and family 5 is 100%, 100%, 85%, 75%, 95%, 85%, and 55%, respectively. All but one of these corresponds to the level of heterozygosity in humans and mice, which was in a range of 80–90% [48]. The sire and dam of family 92 were from family 0751, the sire and dam of family 5 were from family 0750, and sire and dam of family 102 were from family 0768. These exhibited lower heterozygosity (75%, 55%, 85%), especially family 5 with the lowest heteroxygosity (55%), but the survival ratio of family 5 was the highest among the seven families examined in this study. It might indicate that other genes in family 5 or the homozygosity of the MHC class II B gene resulted in the resistance to *V. anguillarum* in the Japanese flounder. Further studies are needed to examine the MHC class II B genes in the offspring of the seven families reported in this study.

Between 5 and 10 clones in each of the individual PCR products had one or seven sequences, and most of these sequences were the same as that of the other clones, indicating that some of these were not the result of PCR amplification “errors” [49] or the mismatch repair of heteroduplex molecules during the course of cloning in *E. coli.* [50]. In this study, ten (20 individuals from family 101 and 104, resp.) or five (10 individuals from family 5, 41, 75, 92 and 102, resp.) clones per individual were sequenced, and we found a significant difference in the allele distribution in the surviving and dead individuals in each of the seven families. It was possible that the results would differ in terms of the clones and samples, so further studies were needed to select a greater number of both for sequencing and analysis.

In summary, the detection of MHC class II B alleles and their polymorphisms as depicted in the present study will be helpful for immunological research in the future. This investigative work has the ultimate aim of developing families or strains of Japanese flounder with bacterial resistance.

## Figures and Tables

**Table 1:** Numbers of the dead/surviving individuals when infected with the *V. anguillarum* were selected from seven families of Japanese flounder.

Family	Individuals per family		Total
	Dead	Surviving	
Family 101	20	20	40
Family 104	20	20	40
Family 5	10	10	20
Family 41	10	10	20
Family 75	10	10	20
Family 92	10	10	20
Family 102	10	10	20

Total	90	90	180

**Table 2:** The alleles and GenBank accession numbers.

Allele	GenBank accession no.	Allele	GenBank accession no.	Allele	GenBank accession no.
Paol-DAB*0103	HQ634973	Paol-DAB*2212	HQ635006	Paol-DAB*4901	HQ635039
Paol-DAB*0104	HQ634974	Paol-DAB*2213	HQ635007	Paol-DAB*5001	HQ635040
Paol-DAB*0105	HQ634975	Paol-DAB*2214	HQ635008	Paol-DAB*5101	HQ635041
Paol-DAB*0106	HQ634976	Paol-DAB*2215	HQ635009	Paol-DAB*5102	HQ635042
Paol-DAB*0107	HQ634977	Paol-DAB*2216	HQ635010	Paol-DAB*5201	HQ635043
Paol-DAB*0108	HQ634978	Paol-DAB*2217	HQ635011	Paol-DAB*5202	HQ635044
Paol-DAB*0109	HQ634979	Paol-DAB*3204	HQ635012	Paol-DAB*5203	HQ635045
Paol-DAB*0110	HQ634980	Paol-DAB*3205	HQ635013	Paol-DAB*5301	HQ635046
Paol-DAB*0111	HQ634981	Paol-DAB*3206	HQ635014	Paol-DAB*5401	HQ635047
Paol-DAB*0112	HQ634982	Paol-DAB*3207	HQ635015	Paol-DAB*5402	HQ635048
Paol-DAB*0113	HQ634983	Paol-DAB*3208	HQ635016	Paol-DAB*5501	HQ635049
Paol-DAB*0114	HQ634984	Paol-DAB*3209	HQ635017	Paol-DAB*5601	HQ635050
Paol-DAB*0115	HQ634985	Paol-DAB*3502	HQ635018	Paol-DAB*5701	HQ635051
Paol-DAB*0116	HQ634986	Paol-DAB*3806	HQ635019	Paol-DAB*5801	HQ635052
Paol-DAB*0117	HQ634987	Paol-DAB*4307	HQ635020	Paol-DAB*5802	HQ635053
Paol-DAB*0118	HQ634988	Paol-DAB*4308	HQ635021	Paol-DAB*5803	HQ635054
Paol-DAB*0119	HQ634989	Paol-DAB*4309	HQ635022	Paol-DAB*5804	HQ635055
Paol-DAB*0120	HQ634990	Paol-DAB*4310	HQ635023	Paol-DAB*5901	HQ635056
Paol-DAB*0121	HQ634991	Paol-DAB*4311	HQ635024	Paol-DAB*6001	HQ635057
Paol-DAB*0122	HQ634992	Paol-DAB*4312	HQ635025	Paol-DAB*6101	HQ635058
Paol-DAB*0123	HQ634993	Paol-DAB*4313	HQ635026	Paol-DAB*6201	HQ635059
Paol-DAB*0304	HQ634994	Paol-DAB*4314	HQ635027	Paol-DAB*6301	HQ635060
Paol-DAB*0802	HQ634995	Paol-DAB*4315	HQ635028	Paol-DAB*6401	HQ635061
Paol-DAB*0902	HQ634996	Paol-DAB*4316	HQ635029	Paol-DAB*6402	HQ635062
Paol-DAB*0903	HQ634997	Paol-DAB*4317	HQ635030	Paol-DAB*6501	HQ635063
Paol-DAB*2204	HQ634998	Paol-DAB*4601	HQ635031	Paol-DAB*6601	HQ635064
Paol-DAB*2205	HQ634999	Paol-DAB*4602	HQ635032	Paol-DAB*6801	HQ635065
Paol-DAB*2206	HQ635000	Paol-DAB*4603	HQ635033	Paol-DAB*6901	HQ635066
Paol-DAB*2207	HQ635001	Paol-DAB*4604	HQ635034	Paol-DAB*7001	HQ635067
Paol-DAB*2208	HQ635002	Paol-DAB*4605	HQ635035	Paol-DAB*7101	HQ635068
Paol-DAB*2209	HQ635003	Paol-DAB*4701	HQ635036	Paol-DAB*7201	HQ635069
Paol-DAB*2210	HQ635004	Paol-DAB*4801	HQ635037	Paol-DAB*7301	HQ635070

Paol-DAB*2211	HQ635005	Paol-DAB*4802	HQ635038	Paol-DAB*6701	HQ635071

**Table 3:** The number of allele in each of seven flounder families.

Individual no.	Allele no.						
	1 allele	2 alleles	3 alleles	4 alleles	5 alleles	6 alleles	7 alleles
Family 101		6	21	7	2	3	1
Family 104		22	8	6	3		
Family 102	3	11	3	3			
Family 92	5	6	8	1			
Family 75	1	9	9	1			
Family 41	3	9	7	1			

Family 5	9	6	5				

Total	25	71	57	18	5	3	1

**Table 4:** The frequency of alleles (>3%) in each of seven families.

Allele	Mode	Number	Frequency	Family	Allele	Mode	Number	Frequency	Family
Paol-DAB*0301	S	21	0.21**	F5	Paol-DAB*4101	S	26	0.26**	F5
	D	38	0.38			D	8	0.08	
	Total	60	0.6			Total	34	0.34	
Paol-DAB*0101	S	12	0.114	F41	Paol-DAB*0801	S	20	0.19	F41
	D	11	0.105			D	29	0.267	
	Total	23	0.219			Total	49	0.467	
Paol-DAB*3803	S	18	0.171**	F41	Paol-DAB*2202	S	5	0.05	F75
	D	6	0.057			D	1	0.01	
	Total	24	0.229			Total	6	0.059	
Paol-DAB*0901	S	12	0.118	F75	Paol-DAB*2201	S	12	0.118	F75
	D	14	0.138			D	11	0.108	
	Total	26	0.255			Total	23	0.226	
Paol-DAB*3805	S	12	0.118	F75	Paol-DAB*0502	S	7	0.069	F75
	D	9	0.088			D	9	0.088	
	Total	21	0.206			Total	16	0.157	
Paol-DAB*3501	S	16	0.158	F92	Paol-DAB*4301	S	25	0.248	F92
	D	25	0.248			D	19	0.188	
	Total	41	0.405			Total	44	0.436	
Paol-DAB*3201	S	24	0.061**	F101	Paol-DAB*0101	S	40	0.102	F101
	D	56	0.142			D	47	0.118	
	Total	80	0.203			Total	87	0.221	
Paol-DAB*2201	S	33	0.084**	F101	Paol-DAB*4301	S	44	0.112**	F101
	D	56	0.142			D	19	0.048	
	Total	89	0.226			Total	63	0.16	
Paol-DAB*0102	S	8	0.02	F101	Paol-DAB*4302	S	12	0.03	F101
	D	9	0.023			D	2	0.005	
	Total	17	0.043			Total	14	0.035	
Paol-DAB*4301	S	30	0.076	F104	Paol-DAB*4601	S	54	0.136**	F104
	D	49	0.123			D	26	0.065	
	Total	79	0.199			Total	80	0.202	
Paol-DAB*3803	S	32	0.081	F104	Paol-DAB*0101	S	43	0.108	F104
	D	33	0.083			D	36	0.091	
	Total	65	0.164			Total	79	0.199	
Paol-DAB*0102	S	16	0.04	F104	Paol-DAB*4302	S	5	0.013	F104
	D	13	0.033			D	13	0.033	
	Total	29	0.073			Total	18	0.045	
Paol-DAB*3804	S	7	0.018	F104	Paol-DAB*3803	S	7	0.072	F102
	D	5	0.013			D	6	0.062	
	Total	12	0.03			Total	13	0.134	
Paol-DAB*0101	S	17	0.175	F102	Paol-DAB*4301	S	12	0.124	F102
	D	21	0.217			D	12	0.124	
	Total	38	0.392			Total	24	0.247	

Notes: S denotes survivor individual and D denotes dead individual in the challenge tests. (One allele Paol-DAB*4601 was first present in this study as well as the other 16 alleles have presented in previous reports [15]). **denotes difference is significant at the 0.05 level (*P* < 0.05).

**Table 5:** Synonymous (*d*_*S*_) and nonsynonymous (*d*_*N*_) substitution rate in the putative peptides binding region (PBR) and nonpeptides binding region (non-PBR) among Japanese flounder families.

Family	Region	No. of codons	*d*_*N*_ (SE)	*d*_*S*_ (SE)	*d*_*N*_/*d*_*S*_
F5	PBR	23	0.134 ± 0.037	0.031 ± 0.022	4.323
	Non-PBR	68	0.04 ± 0.012	0.016 ± 0.009	2.5
	Total	91	0.064 ± 0.014	0.019 ± 0.008	3.368

F41	PBR	23	0.109 ± 0.023	0.048 ± 0.024	2.271
	Non-PBR	68	0.051 ± 0.014	0.046 ± 0.018	1.109
	Total	91	0.066 ± 0.013	0.046 ± 0.014	1.435

F92	PBR	23	0.180 ± 0.034	0.060 ± 0.041	3.000
	Non-PBR	68	0.033 ± 0.010	0.031 ± 0.015	1.065
	Total	91	0.069 ± 0.015	0.037 ± 0.016	1.865

F75	PBR	23	0.167 ± 0.032	0.024 ± 0.013	6.96
	Non-PBR	68	0.047 ± 0.012	0.037 ± 0.018	1.27
	Total	91	0.078 ± 0.013	0.034 ± 0.013	2.294

F102	PBR	23	0.146 ± 0.035	0.009 ± 0.009	16.2
	Non-PBR	68	0.055 ± 0.013	0.039 ± 0.013	1.410
	Total	91	0.079 ± 0.013	0.032 ± 0.013	2.469

F101	PBR	23	0.147 ± 0.030	0.024 ± 0.007	6.125
	Non-PBR	68	0.053 ± 0.013	0.033 ± 0.014	1.606
	Total	91	0.077 ± 0.014	0.031 ± 0.010	2.484

F104	PBR	23	0.177 ± 0.028	0.004 ± 0.004	44.25
	Non-PBR	68	0.050 ± 0.013	0.036 ± 0.014	1.389
	Total	91	0.083 ± 0.014	0.028 ± 0.011	2.964

Whole	PBR	23	0.231 ± 0.051	0.037 ± 0.028	6.243
	Non-PBR	68	0.057 ± 0.020	0.041 ± 0.023	1.390
	Total	91	0.098 ± 0.020	0.038 ± 0.018	2.579
